# The clinical efficacy and mechanism of gamma frequency electroacupuncture stimulation on the rehabilitation of upper limb motor function in stroke patients: study protocol of a randomized clinical trial

**DOI:** 10.3389/fneur.2025.1603522

**Published:** 2025-05-30

**Authors:** Xiaoyu Tian, Xiaoming Yu, Hongli Ma, Minghui Lai, Ruiren Wu, Xinglin Zhang, Jingjing Zhang, Zifan Luo, Wang Fu, Wei Feng, Jun Hu, Chunlei Shan, Cong Wang, Feng Wang

**Affiliations:** ^1^Department of Neurology, Seventh People’s Hospital of Shanghai University of Traditional Chinese Medicine, Shanghai, China; ^2^Engineering Research Center of Traditional Chinese Medicine Intelligent Rehabilitation, Ministry of Education, Shanghai, China; ^3^School of Rehabilitation Science, Shanghai University of Traditional Chinese Medicine, Shanghai, China; ^4^Department of Rehabilitation, Seventh People’s Hospital of Shanghai University of Traditional Chinese Medicine, Shanghai, China; ^5^The Second Rehabilitation Hospital of Shanghai, Shanghai, China; ^6^Department of Rehabilitation Medicine, Tongren Hospital, Shanghai Jiao Tong University School of Medicine, Shanghai, China; ^7^Yuanshen Rehabilitation Institute, Shanghai Jiao Tong University School of Medicine, Shanghai, China; ^8^Queensland Brain Institute, The University of Queensland, Saint Lucia, QLD, Australia

**Keywords:** electroacupuncture stimulation, gamma oscillations, stroke, upper limb motor function, neurological recovery

## Abstract

**Background:**

The field of neuroscience has discovered that gamma oscillations (30–100 Hz) in the brain play a key role in neuroplasticity, information conduction and neuroprotective mechanisms. Electroacupuncture stimulation (ES) is a modern treatment method that combines the advantages of Chinese acupuncture with those of electrical stimulation, and is widely used in the field of stroke rehabilitation. At present, there is a lack of research on the clinical efficacy and mechanism of applying gamma frequency (40-Hz) ES, a new neuromodulation mode of integrated Chinese and Western medicine, to induce gamma oscillations and promote the rehabilitation of upper limb motor function in stroke patients.

**Methods and analysis:**

This trial uses a randomized, double-blind and controlled method to evaluate the effect of 40-Hz ES of LI11 (Quchi) acupoint on upper limb motor functional rehabilitation in stroke patients. Based on this new rehabilitation program combining Traditional Chinese medicine with modern technique, all patients will receive baseline assessment, 1-week post-intervention assessment and 2-week post-intervention assessment. The primary outcomes are the Fugl-Meyer Assessment Upper Extremity Scale. The secondary indicators include electroencephalogram, National Institutes of Health Stroke Scale, Mini-mental State Examination and Modified Barthel Index.

**Discussion:**

This trial offers novel perspectives on the application of 40-Hz electroacupuncture stimulation in neural oscillation regulation and the effectiveness of this clinically widespread technique in treating upper extremity dysfunction after a stroke and improving patients’ quality of life. The results of this study will contribute to the scientific community’s understanding of the potential mechanisms of this novel neuromodulation method, including changes in neural network connectivity, and improve existing clinical treatments to benefit more stroke patients.

**Clinical trial registration:**

https://www.chictr.org.cn/showprojEN.html?proj=217786, identifier ChiCTR2400082208.

## 1 Introduction

Stroke is the damage of the brain in molecular cells, neural loops and brain network connection caused by the abnormal blood supply caused by the obstruction or rupture of brain blood vessels (1). The total number of prevalent strokes, deaths, and disability-adjusted life years due to stroke increased steadily from 1990 (2). Stroke remains the second-leading cause of death and the third-leading cause of death and disability combined in the world (3). In the context of the demographic development with an aging population, the number of stroke patients is expected to increase significantly in the coming years (4). Motor impairment after stroke typically affects about 80% of patients (5). The incidence of upper limb motor dysfunction in stroke patients is as high as 55 to 75%, and more than one third of persistent upper limb motor dysfunction remained in patients even 6 months after onset (6). This dysfunction is mainly manifested as decreased muscle strength, increased muscle tension and abnormal movement mode, which often causes limb swelling, pain and other discomfort, seriously affects the daily life of patients, and brings great physical and psychological burden to patients and their families (7). Clinical trials have shown that routine rehabilitation intervention is not significant in improving the neurological recovery after stroke, so it is a top priority to seek new stroke rehabilitation therapy (8).

Studies have proved that stimulating the brain is an effective neuromodulation means to promote post-stroke rehabilitation and brain function recovery (9). In recent years, sensory stimulation (such as optical flicker stimulation, sound stimulation, tactile stimulation, etc.) has been proved to be a new non-invasive Neuromodulation technique with clinical application prospects, which has positive significance for improving the rehabilitation status of stroke patients (10–14).

Gamma oscillations (30–100 Hz), a synchronous neural activity common in many brain regions, are thought to be a functional feature of brain networks (15–18). This functional characteristic of the brain will change after stroke (19). Gamma oscillations have been demonstrated to promote neuroplasticity and neuroprotection by regulating synaptic connections and reducing neuronal damage with inflammatory responses in microglia (1, 20). Studies have proved that gamma oscillations enhance synaptic strength and modify neural network connections, promoting neuroplasticity and facilitating the reorganization and repair of damaged neural pathways in the brain, which underpins the recovery of upper limb motor function (21). Additionally, a recent research has demonstrated a significant positive correlation between increased gamma power and the FMA-UE scores of moderate to severe stroke patients during the recovery period (22). Inducing gamma oscillations has shown significant potential in promoting upper limb motor function recovery post-stroke, offering novel strategies and approaches for neurorehabilitation.

Previous trials have proved that a specific frequency of sensory stimulation as a neuromodulation technique, can provide a more comprehensive stimulation, activate sensory receptors induce brain gamma oscillation, activate the damaged nervous system, promote nerve recovery, by increasing the cerebral cortex excitability and arousal, improve the ischemic blood supply stimulation nerve function reconstruction (23–25). Sensory stimulation therapy has a low risk and fewer side effects, and currently there are only reports of using 40-Hz sensory stimulation to stimulate gamma oscillations to promote the recovery of cognitive function in Alzheimer’s disease mouse model or patients (26–29). There is a lack of research on the clinical efficacy and mechanism of applying 40-Hz sensory stimulation to induce gamma oscillations and promote the rehabilitation of upper limb motor function in stroke patients.

Electroacupuncture stimulation (ES), a modern treatment method that combines the advantages of acupuncture in traditional Chinese medicine with the advantages of electrical stimulation (30, 31), is selected as the stimulation method for this trial. ES produces physical stimuli at specific acupuncture points that are detected by sensory receptors, whose activation triggers peripheral sensory neurons to transmit signals to the spinal cord, where the sensory information is processed locally to elicit reflexes and is then transmitted to the brain for nervous regulation (32–34). ES as a form of sensory input, has been shown to correct abnormal brain activity by having the ability to alter the frequency, amplitude, and synchronization patterns of neural oscillations, thereby promoting brain function and aiding in neurological rehabilitation (27, 31). Studies have proved that electroacupuncture stimulation of LI11 can reduce neurological deficits and cerebral infarction volume, inhibit neuronal apoptosis and autophagy, and exert neuroprotective effects (35–37). Previous trials have proved that ES can effectively improve the motor function of the upper limbs in stroke patients (38).

Studies have proved that five major different mechanisms are involved in the beneficial effects of ES on ischemic stroke rehabilitation: Promotion of neurogenesis and cell proliferation in the central nervous system, Regulation of cerebral blood flow in the ischemic area, Anti-apoptosis in the ischemic area, Regulation of neurochemicals, Improvement of impaired long-term potentiation and memory after stroke (39). While traditional electroacupuncture protocols have demonstrated therapeutic efficacy in stroke rehabilitation, they also have limitations and do not fully consider the significance of employing specific frequencies of electrical stimulation. For instance, it remains to be determined whether 40-Hz ES can directly induce gamma oscillations in the brain and thereby harness the unique therapeutic effects of gamma oscillations in neurorehabilitation. The potential of 40-Hz ES on the rehabilitation of upper limb motor function in stroke patients is an area that warrants further investigation. The overall objective of this randomized clinical trial is to seek the clinical efficacy and mechanism of 40-Hz ES to promote upper limb motor functional rehabilitation of stroke patients from the levels of rehabilitation efficacy, neural circuit and brain network.

## 2 Materials and methods

### 2.1 Study design

**Figure 1:**
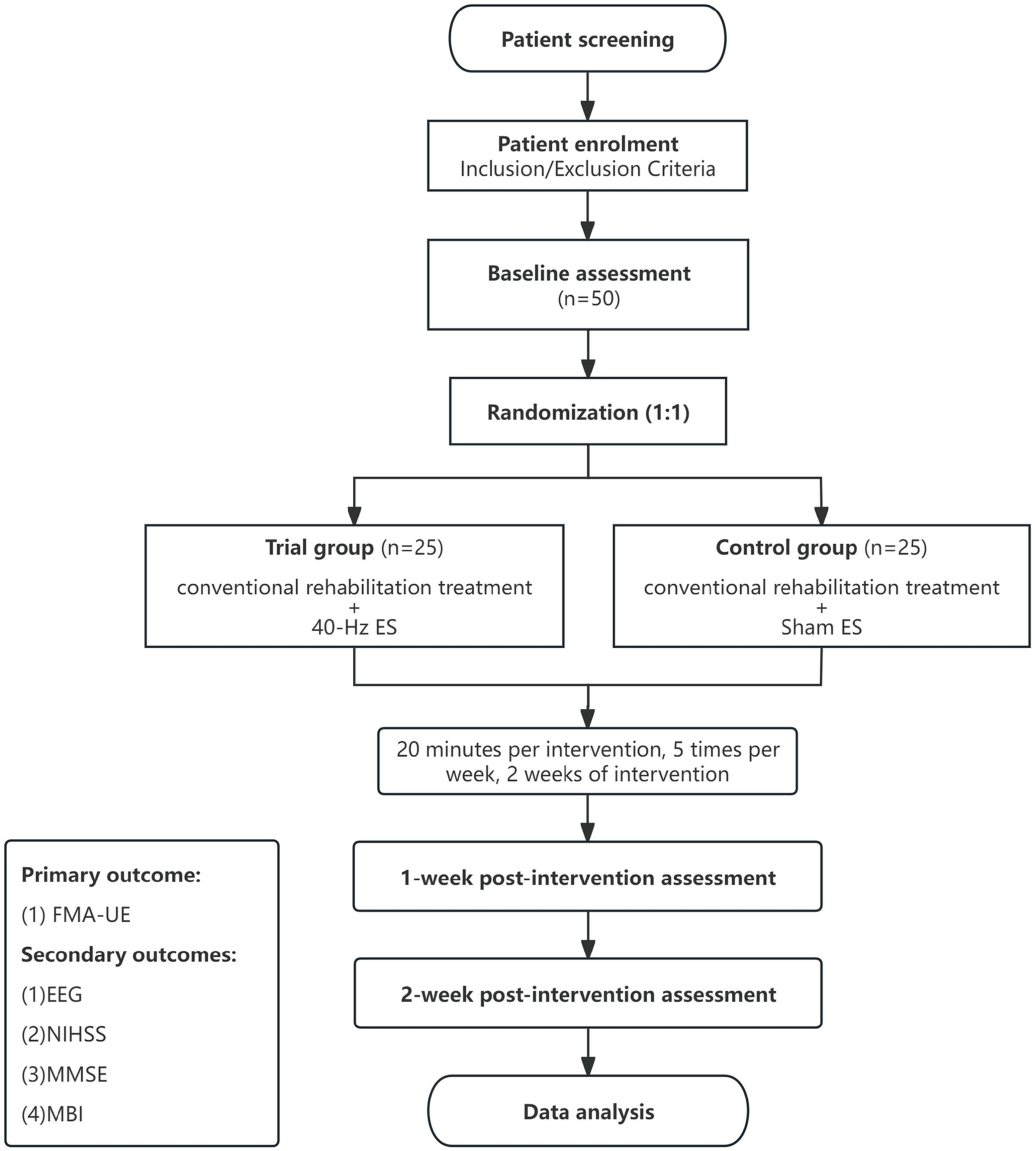
Flow chart of the study. FMA-UE, Fugl-Meyer Assessment Upper Extremity Scale; EEG, Electroencephalogram; NIHSS, National Institutes of Health Stroke Scale; MMSE, Mini-mental State Examination; MBI, Modified Barthel Index.

The study is a prospective, randomized, double-blind, exploratory, controlled trial design study. In this trial, 50 stroke hospitalized patients who meet the inclusion criteria will be randomly divided into trial group (*n* = 25) and control group (*n* = 25) at a ratio of 1∶1. The patients in the trial group will be given conventional rehabilitation treatment and 40-Hz continuous wave ES on LI11 of the affected upper limb (20 min per intervention, 5 times per week), and the intervention will last 2 weeks as shown in Figure 1. The patients in the control group will be given conventional rehabilitation treatment and sham ES on LI11 of the affected upper limb (20 min per intervention, 5 times per week), and the intervention will last 2 weeks as shown in Figure 1.

### 2.2 Sample size

According to the previous literature, the clinically important difference of the FMA-UE scores ranged from 4.25 to 7.25 points, and the improvement of FMA-UE score of 5.25 marks a significant improvement in upper limb motor function (40). Let *α* = 0.05 (bilateral), the power at 0.80. According software, *n* = 20 can be obtained, and with a drop-out rate of around 20%, the sample size required for each group is 25 cases each. Furthermore, we will establish robust communication protocols during the study to minimize participant dropout and ensure adequate final sample size. Before the study begins, we will thoroughly explain its importance and significance to participants, fostering their comprehension and willingness to cooperate. Throughout the study, we will promptly address any questions or concerns that arise, maintaining participant engagement.

### 2.3 Randomization

The type of randomization that will be taken for this trial is simple randomization. 50 patients with upper limb motor dysfunction of stroke who met the inclusion criteria will be numbered as 1–50 according to the enrolment order, and the random number table generated by the Microsoft Excel random function will be used to sort and group according to the random number generated, and the patients will be randomly included in the control group and the trial group, with 25 cases in each group.

The transparent sealed envelopes (each stratified with a randomization list) will be prepared by an opaque sealed envelope with a serial number (each stratified randomization list) prepared by an independent statistician and sent to the clinical trial center. Independent investigators will distribute the envelope to participants and assign participants to the study group in a 1:1 ratio without stratification. Before randomization, all participants will be informed that they will be assigned to one of the two groups. Allocation results will not be released to participants until the end of the trial data collection phase. The allocation results will be released to the participants only after the data collection phase of the trial. The investigators who collect and analyze the data related to this trial will be blinded to the participant allocation outcomes.

### 2.4 Blinding

Subjects are blind when receiving the group. All participants were thoroughly informed that the intensity of electrical stimulation administered during the trial might fall below the conventional threshold of human sensory perception. Within the context of the treatment protocol, it is important to note that, although the power indicator of the electroacupuncture apparatus used in the control group was illuminated, the device was carefully configured to ensure that no electrical current was actually delivered to the participants. Investigators who perform ES, clinical assessment, and data analysis will set blind spots for group assignment. Randomization is performed by an independent biostatistician. The biostatistician is the only one who has access to check the file. The allocation list is kept in a separate file on a separate computer under password protection. Since the study code for each participant will not be associated with the group assignment, the person performing the data analysis will not be able to identify the subject’s study group. The current intensity adjustment based on patient tolerance will be executed by an investigator who is distinct from the one responsible for follow - up assessment. The evaluator will remain blinded to the patients’ group assignments. By decoupling the operator from the evaluator, we preclude the evaluator from inferring patient group allocation through procedural information. Moreover, during data recording and analysis, strict anonymity will be preserved for all data, thereby fortifying the blinding technique’s effectiveness.

### 2.5 Unblinding

Staff members who perform the ES and the biostatistician who performs the allocation are not blind. Data will be unblinded when data analysis is completed and statistical tests between study groups need to be performed.

### 2.6 Inclusion/exclusion criteria

The inclusion criteria for the participants are the following:

1. Meet the criteria for ischemic stroke in the Chinese Guidelines for the Diagnosis and Treatment of Acute Ischemic Stroke 2023, and confirmed by CT or MRI of the head.
2. The patient had a first-time stroke with unilateral hemiplegia.
3. Onset ranged from 2 weeks to 3 months, and vital signs were stable.
4. Age 45–80 years old.
5. The patient had upper limb motor dysfunction in clinical manifestations, and FMA-UE scores in the 0–30 range.
6. The patient can sit independently for 1 h or more.
7. Patients who are in stable condition, conscious, have no aphasia or disorder, can understand the content of the scale, and cooperate with examination and treatment.
8. The patient has not taken any sedative medication or muscle relaxant for 2 weeks.
9. Informed consent to this trial, and signed written informed consent.

The exclusion criteria for the participants are the following:

1. Patients who are critically ill or in the acute phase and whose condition has not yet stabilized.
2. Patients with deafness, aphasia, or severe cognitive impairment that make it difficult to communicate normally.
3. Patients with systolic blood pressure > 180 mmHg or diastolic blood pressure > 110 mmHg, patients with severe cardiovascular, liver, kidney, blood, digestive, respiratory and other primary diseases, patients with mental illness, malignant tumors, venous thrombosis, osteoporosis, severe bleeding tendency and infection at the treatment site.
4. Patients who are unable to recover with the study protocol or participate in other clinical experiments within 3 months or receive other related treatments in the middle of the study, which may affect the efficacy judgment of this study.
5. Dystonia and previous motor dysfunction caused by other reasons.
6. Pregnant and lactating women.
7. Hemiplegia upper limb with previous trauma and bone and joint diseases.
8. The patient is in a state of severe spasticity, i.e., modified Ashworth scale above grade IIII.
9. The patient has pacemakers, cochlear implants and other instruments and equipment in the body.
10. The patient has had vagus nerve surgery.
11. The patient has sensory disturbances of the skin.

Data integrity is compromised and data will not be included in the analyses when the following:

1. The patient voluntarily requested to withdraw.
2. The patient is concurrently involved in other similar therapeutic activities.
3. The patient compliance with the experiment was poor.
4. The patient develops other conditions or serious adverse events that affect the experiment.

### 2.7 Study setting

The study will be conducted in the Seventh People’s Hospital of Shanghai University of Traditional Chinese Medicine. All participants are given a unique identifying number that is used on all clinical trial data for that participant. The database will be constructed using Excel (Microsoft, USA 2022 version), and regular data monitoring will be undertaken in accordance with the sponsor’s standard operating procedures. Clinical data in paper format are kept in a locked file cabinet, while clinical data entered directly from the source documents along with the trial data are saved with a unique code in password-protected computers.

### 2.8 Recruitment

This clinical study will be conducted at the same location with participants without blind spots for treatment task assignment. This study is aimed at patients diagnosed with stroke and associated with upper limb motor dysfunction in the Seventh People’s Hospital of Shanghai University of Traditional Chinese Medicine. Participants will be recruited by distributing pamphlets, websites, and ads on social media. To ensure effective recruitment, research team members will assist in contacting potential participants. Demographic information for each participant will be collected at the first appointment (enrollment). Participants will not be limited by age, gender, or ethnicity. Eligible participants will be informed about the study procedures and any further arrangements are required to be made. Recruitment will be stopped when the number of patients reaches the number of patients expected.

### 2.9 Participant timeline

**Figure 2:**
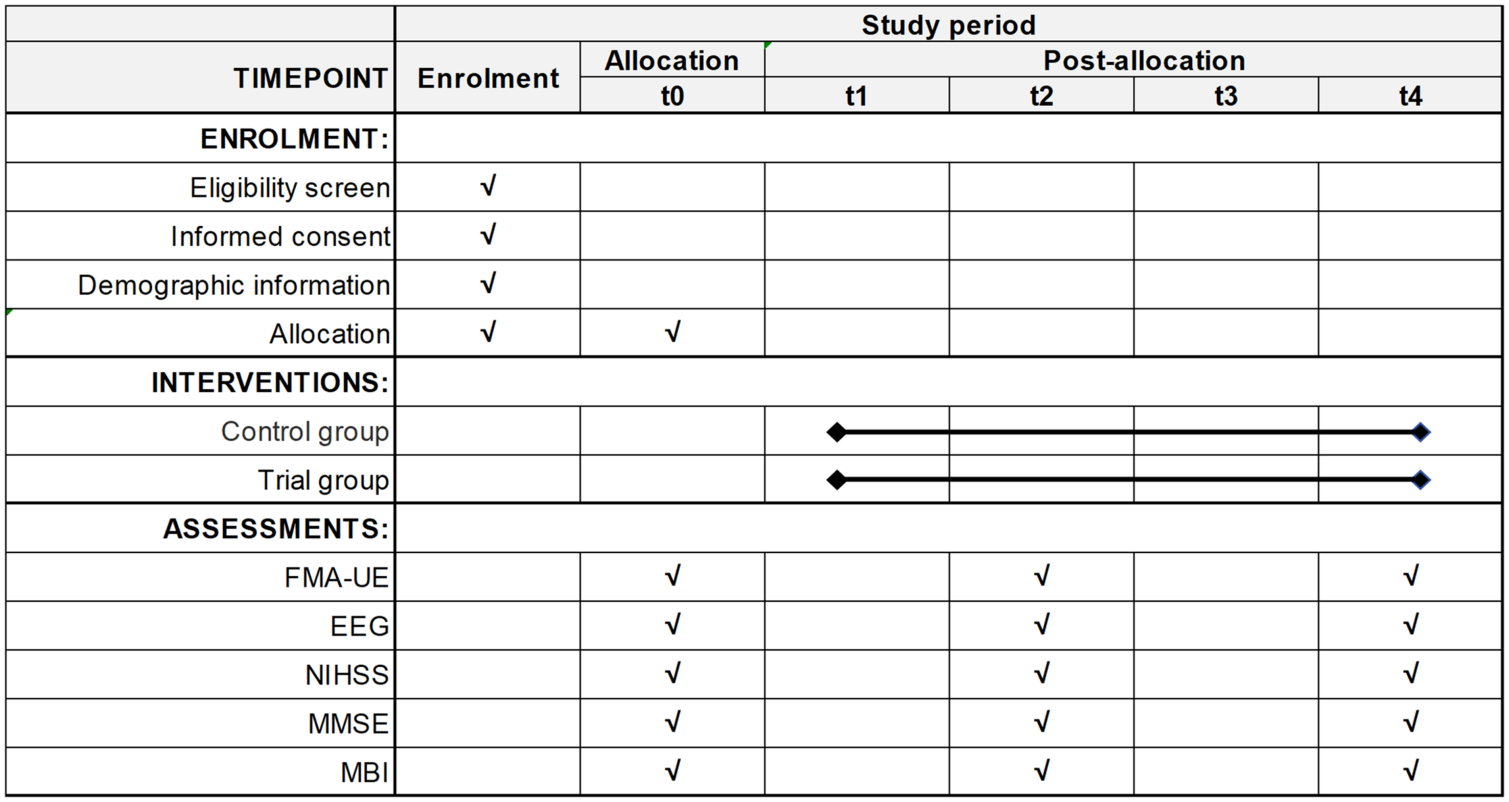
Schedule of enrollment, interventions, and assessments. “√” means things will be done. t0: Baseline assessment. t1: The first week of the intervention. t2: 1-week post-intervention assessment. t3: The second week of the intervention. t4: 2-week post-intervention assessment.

The participant timeline is shown in Figure 2. Participants who meet the screening criteria will undergo a “Baseline assessment” and complete an informed consent form and randomization. Participants in the trial group will undergo conventional rehabilitation treatment over a two-week period and complete 10 ES interventions. Control group participants will undergo conventional rehabilitation treatment over a two-week period and complete 10 sham ES interventions. Conventional rehabilitation treatment include proper limb positioning, transfer training, range of motion exercises, standing training, walking training, aerobic exercise, progressive resistance training, task-specific training, constraint-induced movement therapy, balance training, and mirror therapy (41). The 1-week post-intervention assessment will be conducted at the fifth intervention and the 2-week post-intervention assessment will be conducted at the tenth intervention.

### 2.10 Informed consent

Trained and experienced clinical professionals will be responsible for obtaining informed consent from potential participants. Throughout this process, the research team will ensure that each potential participant comprehensively understands the study’s purpose, methodologies, potential risks and benefits, as well as the possible effects of participation on their health and overall well-being. The clinical professional will provide a thorough explanation of the informed consent form’s content, ensuring that participants can ask pertinent questions and receive satisfactory responses. Additionally, participants will be explicitly informed of their right to withdraw from the study at any time without experiencing any negative repercussions. This approach aims to uphold the autonomy of participants and guarantee that they possess adequate information and understanding when making decisions regarding their participation.

### 2.11 Additional consent provisions for collection and use of participant data and biological specimens

No biological specimens are collected for research purposes.

### 2.12 Interventions

#### 2.12.1 Trial group

All participants were thoroughly informed that the intensity of electrical stimulation administered during the trial might fall below the conventional threshold of human sensory perception and that some may not feel the electrical stimulation. 40-Hz ES will be given at the affected side LI11 (quchi) of the patient, with an intensity starting at 0 mA and adding 0.1 mA each time to avoid pain to the patient tolerance (When the patient develops pain, immediately stop continuing to increase the current and drop 0.1 mA).

#### 2.12.2 Control group

All participants were thoroughly informed that the intensity of electrical stimulation administered during the trial might fall below the conventional threshold of human sensory perception and that some may not feel the electrical stimulation. Sham ES will be given at the affected side LI11 (quchi) of the patient, although the power indicator of the electroacupuncture apparatus used in the control group was illuminated, the device was carefully configured to ensure that no electrical current was actually delivered to the participants.

#### 2.12.3 Intervention description

**Figure 3:**
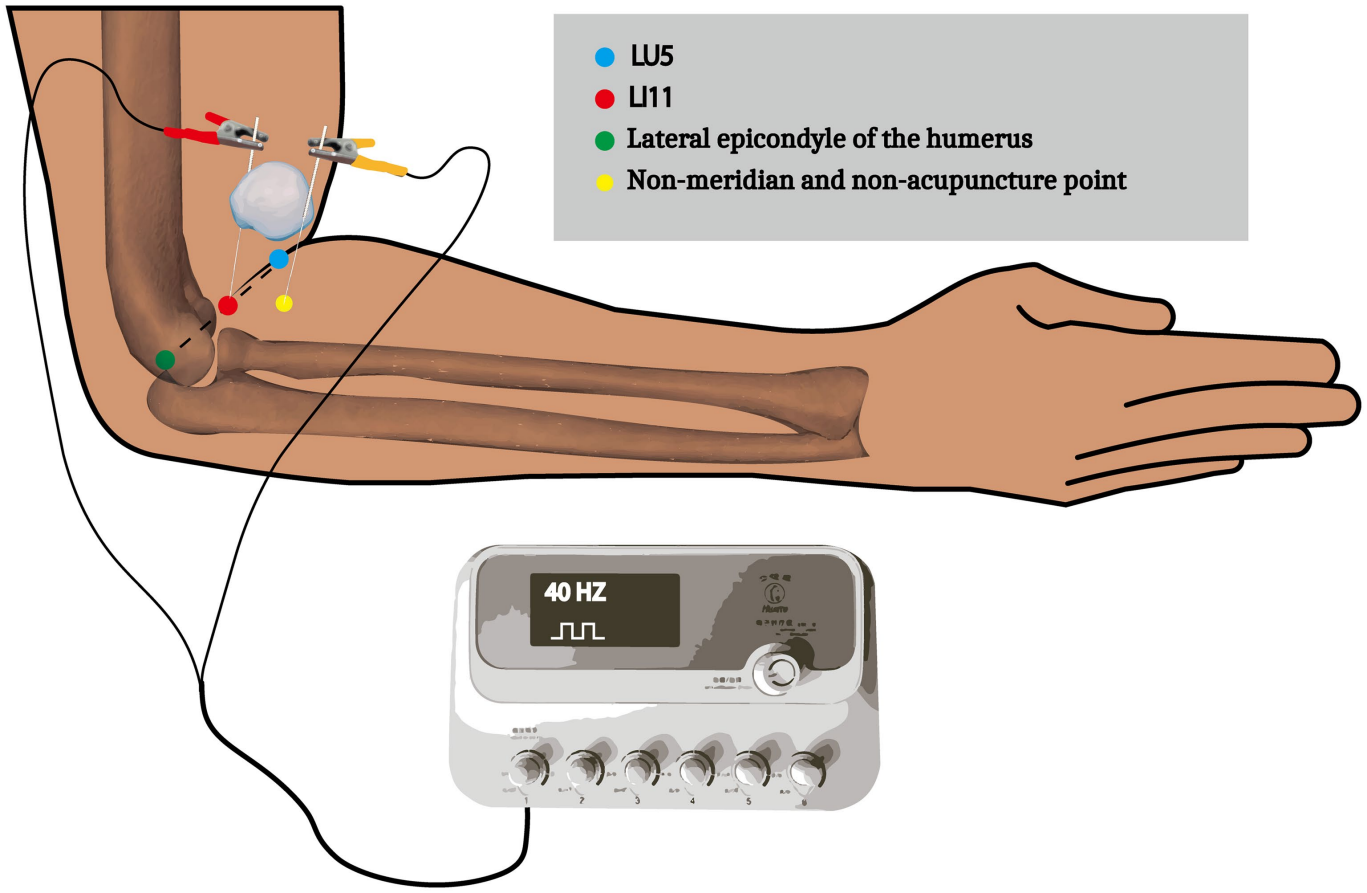
A schematic diagram of the acupuncture intervention. LU5 belongs to the Hand Taiyin Lung Meridian, which is located in the elbow area, on the transverse stripe of the elbow, in the concave radial margin of the biceps brachii tendon. LI11 belongs to the Hand Yangming Large Intestine Meridian and is located at the lateral end of the transverse elbow stripe, with the elbow flexed, at the midpoint of the line connecting the LU5 and the Lateral epicondyle of the humerus. Place a dry sterile cotton ball between the two needles to prevent short circuits caused by accidental contact between the cathode and anode.

The acupuncture intervention is shown in Figure 3. Use disposable sterile acupuncture needles (0.30 × 25 mm, Hwato, Suzhou Medical Supplies Factory Co., Ltd., China) to puncture LI11 and a non-meridian and non-acupuncture point adjacent to LI11. The precise location of the LI11 is the midpoint of the line connecting the LU5 and the lateral epicondyle of the humerus. The location of the LU5 and the lateral epicondyle of the humerus can be directly localized by the body surface structures. Use the electroacupuncture stimulation treatment instrument (Hwato SDZ-III, Suzhou Medical Supplies Factory Co., Ltd., China) to output 40-Hz bidirectional rectangular pulse current. Before using the electroacupuncture stimulation treatment instrument, adjust the intensity adjustment knob to 0 (no output), adjust the frequency adjustment knob to 40-Hz, set the time to 20 min, and then connect the two pairs of output electrodes on the machine to two needles, with an intensity starting at 0 mA and adding 0.1 mA each time to avoid pain to the patient tolerance (When the patient develops pain, immediately stop continuing to increase the current and drop 0.1 mA). At the end of the intervention, adjust the intensity adjustment knob to 0 (no output), the power should be turned off, and the electrode clamp should be removed from the needle shank and the needle inserted into the tissue should be removed. The intervention will be performed 5 times a week.

#### 2.12.4 Criteria for discontinuing interventions

If the patient develops other medical conditions during the intervention that interfere with the intervention, the intervention should be discontinued immediately and the circumstances documented. If the patient has participated in the assessment and intervention for more than 2 weeks, the patient’s data should still be recorded and counted; if the patient initiates discontinuation of the intervention during the intervention, the intervention should be discontinued immediately and the reason for discontinuation should be recorded in a timely manner.

Interventions will be halted if unanticipated problems (UPs) or serious adverse events (SAEs) arise that raise concerns about the intervention’s safety. Additionally, if new information emerges during the trial that warrants discontinuation, the trial will be stopped accordingly. Currently, numerous published studies have utilized 40-Hz sensory stimulation to evoke gamma oscillations in healthy subjects and Alzheimer’s patients, and no serious adverse effects have been reported. Our group is conducting a clinical trial of 40-Hz sensory stimulation induced gamma oscillations for stroke rehabilitation, and no adverse events have occurred.

#### 2.12.5 Strategies to improve adherence to interventions

Before the implementation of ES of patients, patiently explain the specific operation of acupuncture to patients in detail, so that patients can understand the safety of this intervention, so as to win the trust and cooperation of patients.

#### 2.12.6 Relevant concomitant care permitted or prohibited during the trial

During the trial period, registered participants are prohibited from enrolling in other clinical trials. The research team will provide periodic reminders to participants to refrain from initiating any additional rehabilitation programs throughout the study duration. This approach is essential to maintain the validity and reliability of the research findings.

#### 2.12.7 Provisions for post-trial care

Should participants incur any harm as a consequence of the trial, they will be provided with treatment in accordance with established medical protocols.

### 2.13 Outcomes

All patients will receive baseline assessment, 1-week post-intervention assessment and 2-week post-intervention assessment. The primary outcome is the FMA-UE. The secondary indicators include EEG, NIHSS, MMSE and MBI.

#### 2.13.1 FMA-UE

FMA-UE is primarily used to evaluate reflex activity, motor control, and muscle strength in the hemiplegic upper extremity, and it is widely used as a rehabilitation test and to record outcomes of recovery after stroke. It can make accurate quantitative assessment of the limb function of hemiplegia stroke patients, and the assessment is more comprehensive, which can intuitively and quickly reflect the abnormal movement patterns of patients (42). The FMA-UE includes a total of 33 upper limb proximal and distal limb movement-related items, with a total score of 66. The scoring criteria are as follows: each item is scored on a scale of 0, 1, and 2, with 0 being unable to complete at all, 1 being able to complete only part of the activity, and 2 being able to complete the prescribed movements, with higher scores and better motor function. Prior to each assessment, participants will be allowed to practice using their unaffected side to avoid learning effects. Studies have proved that the clinically important difference of the FMA-UE scores ranged from 4.25 to 7.25 points, and the improvement of FMA-UE score of 5.25 marks a significant improvement in upper limb motor function (40). We defined a clinically meaningful response as a 5.25-point in FMA-UE score based on previous research (43).

#### 2.13.2 EEG

The EEG data will be collected through a 32-lead wireless EEG acquisition system (iRecorder, Shanghai Niantong Intelligent Technology Co., Ltd., China). A tranquil and electrically shielded examination room will be employed for the study. Participants will be instructed to remain awake and quiet while seated comfortably, refraining from engaging in any cognitive or mental tasks. All assessments will be conducted by a single EEG examiner. The system will possess a common-mode rejection ratio of 130 dB, an input impedance of 200 GΩ, and an input noise level of less than 0.2 μVrms, with a resolution of 24 bits. The sampling frequency will be established at 1000 Hz, and the impedance for each channel will be maintained below 20 kΩ. The data processing and analysis software Matlab2024a of THE MATH WORKS company and EEGLAB toolbox will be used to analyze EEG data offline (44). The reference electrode will be converted from the recorded Cz electrode to the average of all electrodes (45). Independent component analysis will be performed, and artifacts will be removed using the ADJUST algorithm (46). We perform spectral analysis on EEG data collected during resting state using the Hamming window and Fast Fourier Transform (FFT). Through FFT transformation, we analyze the absolute power of each frequency band, thereby elucidating the characteristics of EEG activity across different frequency ranges. The resting state electroencephalogram data are further analyzed using functional connectivity (FC) techniques. We employ filtering methods to remove power frequency interference and electromyographic noise, followed by the application of bandpass filters to extract gamma band signals. After filtering, we utilize the Hilbert transform to process the filtered signal from each electrode, which enables us to extract phase information. Subsequently, we calculate coherence as a measure of signal similarity between electrodes within the gamma band. Based on these calculations, we construct a connection matrix to visualize the connection strength and patterns among different electrodes using a network diagram. This comprehensive analytical approach reveals the characteristics and activity patterns of brain networks under the influence of 40-Hz ES. We will explore the relationship between changes in EEG parameters and other clinical outcomes to gain a more comprehensive understanding of the neurophysiological mechanisms underlying the effects of ES.

#### 2.13.3 NIHSS

The NIHSS is now considered the primary tool for evaluating neurological changes in patients with acute ischemic stroke and for determining treatment options and predicting patient outcomes (47–49). The NIHSS Neurological Check consists of a comprehensive assessment comprising 11 items. These items assessment various neurological functions, including the level of consciousness—assessed through three components (level of consciousness, responses to questions, and execution of commands)—as well as best gaze, visual function, facial palsy, motor function of the arms and legs, limb ataxia, sensory perception, best language capability, dysarthria, and the presence of extinction or inattention. It shows good correlation with the volume of cerebral infarction measured by brain computed tomography (scan) at 7 days and also shows good predictive correlation of 3-month outcome (50, 51). The NIHSS possesses good interrater reliability (mean kappa = 0.69) and test–retest reliability when performed by neurologists and nonneurologists (mean kappa = 0.66–0.77) (52). Correlation analysis of FMA-UE scores with NIHSS scores will help us to determine whether improvement in neurological deficits correlates with recovery of upper limb motor function.

#### 2.13.4 MMSE

The MMSE is a widely recognized and extensively utilized screening instrument for the assessment of cognitive impairment. This 30-point questionnaire provides a comprehensive evaluation of an individual’s cognitive capacities, encompassing a diverse array of functions such as orientation, memory, attention, language, and visual–spatial skills. A score of 24 or higher is generally considered within the normal range, whereas a score falling below this threshold may serve as an indicator of potential cognitive impairment. The MMSE takes around 10–15 min to administer and is performed by a healthcare professional. The MMSE has limitations, such as being influenced by education level, age, and language factors. We shall employ the MMSE in tandem with additional assessments, such as the EEG, to facilitate a more comprehensive evaluation of cognitive function (53). We will explore the impact of changes in cognitive function on motor function through variations in MMSE scores and FMA-UE scores.

#### 2.13.5 MBI

The MBI score is used to assess the ability to perform activities of daily living (54), including 10 functions, urination control, modification, toilet use, eating, bed chair transfer, bathing, walking, dressing and walking up and down stairs. Full score = 100 points. Higher scores indicate better independence, and lower scores indicate stronger dependence (55). The MBI score and the FMA-UE score complement each other, reflecting the patient’s recovery status from different dimensions, which can intuitively reflect the clinical efficacy of ES.

### 2.14 Data statement

#### 2.14.1 Plans for assessment and collection of outcomes

Scale assessments will be executed by qualified rehabilitation physicians in collaboration with researchers (S1, S2, S3, S4). To improve the reliability of the findings, specific tests will be administered three times, with the results subsequently averaged. EEG recordings will be collected by the same investigator employing uniform equipment and settings to ensure consistency and repeatability across assessments. Furthermore, the data from each participant will be subjected to validity checks to verify completeness.

#### 2.14.2 Plans for collection, laboratory evaluation and storage of biological specimens for genetic or molecular analysis in this trial/future use

There will be no biological specimens collected during the entire trial.

#### 2.14.3 Data retention

Throughout the study, participants’ personal information will be safeguarded with the utmost confidentiality and will be accessible exclusively to authorized personnel. Each participant will receive a unique trial identification number, and their information will be securely stored within a password-protected database at the clinical trial center. Upon the conclusion of the clinical trial, all data pertaining to participants will be anonymized prior to analysis. The project will strictly comply with all relevant laws and regulations, retaining comprehensive documentation related to the clinical trial—including the trial protocol, approval documents from the Ethics Committee, summary reports, case report forms, contracts, records of serious adverse reactions and events, third-party records, and original trial documents—for a minimum of 3 years after the trial’s completion. Clinical data in paper format will be securely stored in a locked filing cabinet, while electronic data captured directly from source files will be housed on a password-protected computer with a unique access code. All research data will be subjected to statistical analysis and will only be published in a scientific paper following the acquisition of informed consent from participants, who will be queried prior to enrollment regarding their permission to disclose research data in publication (excluding details concerning disease onset, age, and gender).

#### 2.14.4 Availability of data and materials

The principal investigators, Feng Wang, Cong Wang and Chunlei Shan, will have access to the final trial dataset. Data can be provided to researchers upon request, subject to a review of privacy. Requests for data can be sent to the corresponding author by email.

### 2.15 Statistical methods

#### 2.15.1 Statistical methods for primary and secondary outcomes

We will use SPSS 23.0 statistical software for statistical analysis. The measurement data with normal distribution and homogeneity of variance will be expressed as ($\bar{x}$ ± s). If the test of variance is homogeneous and all samples follow normal distribution, the *t*-test will be selected; if the variance is uneven or a sample does not follow normal distribution, a Chi-square test will be used. If the results of ANOVA or Chi-square test are significant, further multiple comparisons will be needed, such as the SNK method, LSD method, extended *t*-test. *p* < 0.05 will be considered as statistically significant.

We will conduct Pearson’s correlation analyses to precisely quantify the relationships between changes in FMA-UE scores and changes in key EEG parameters, specifically focusing on gamma - band power and functional connectivity. These analyses will allow us to determine the extent to which electroacupuncture - induced modulation of brain activity, as reflected in EEG measures, is associated with improvements in upper limb motor function. We will also explore the relationship between changes in EEG parameters and other clinical outcomes to gain a more comprehensive understanding of the neurophysiological mechanisms underlying the effects of electroacupuncture.

We will utilize mediation and moderation analyses to dissect the complex interplay between neurological impairment (NIHSS), cognitive function (MMSE), daily living activities (MBI), and the effects of electroacupuncture on motor recovery. Specifically, we will investigate:

1. Whether improvements in cognitive function (MMSE) or daily living activities (MBI) mediate the relationship between electroacupuncture and gains in motor function (FMA - UE), suggesting that electroacupuncture’s effects on motor function are partially mediated by cognitive or functional improvements.
2. Whether pre - existing levels of neurological impairment (NIHSS), cognitive function (MMSE), or daily living activities (MBI) moderate the effect of electroacupuncture on motor recovery, influencing the magnitude of the treatment response.

Moreover, we will explore potential bidirectional relationships between these variables and their combined impact on motor recovery. For instance, we will examine whether improvements in motor function (FMA - UE) may also influence cognitive function (MMSE) or daily living activities (MBI), and whether these changes in turn further enhance motor recovery. This comprehensive approach will allow us to better understand the dynamic interactions between different aspects of recovery and how they collectively contribute to the overall effectiveness of electroacupuncture.

#### 2.15.2 Methods in analysis to handle protocol non-adherence and any statistical methods to handle missing data

If patients have adverse reactions and discomfort during the trial, the trial will be stopped immediately and relevant information will be recorded. If the patient has been involved in the evaluation and treatment for more than 2 weeks, the data of the patient should still be recorded and counted. If the patient offers to suspend the trial, he should immediately stop the trial and record the reason for the suspension in time. We will conduct an Intention-to-Treat analysis, incorporating all randomly assigned participants, regardless of whether they withdrew prematurely. This approach maintains the original random allocation, minimizes selection bias, and preserves the sample’s randomness.

#### 2.15.3 Methods for additional analyses

Subgroup analyses are not planned for this study.

### 2.16 Oversight and monitoring

#### 2.16.1 Composition of the coordinating center and trial steering committee

The Trial Leader and the Study Director will form the Steering Committee (SC). The SC manages the entire project and has the ultimate authority over the research. The SC will write and submit the report for publication at the end of the study.

#### 2.16.2 Composition of the data monitoring committee, its role and reporting structure

An independent Data Safety Monitoring Board (DSMB) has been established for this study. All members are required to be free of any conflicts of interest related to the research project and its investigators, in alignment with the policies set forth by the National Institute of Mental Health (NIMH). The study database will be created using Microsoft Excel (2022 version), and regular data monitoring will be conducted in accordance with the standard operating procedures. This monitoring will adhere to the Technical Guidelines for Clinical Trial Data Management established by the Chinese National Medical Products Administration.

### 2.17 Adverse event reporting and harms

Adverse events (AEs) serious unexpected adverse events (SUAEs), UPs, or SAEs reported by participants or observed by researchers are recorded. All events are documented and SAEs, SUAEs, and UPs are immediately reported to the DSMB and SC.

### 2.18 Frequency and plans for auditing trial conduct

The trial will be conducted and monitored by the principal investigator. The SC will meet weekly to review and evaluate updates.

### 2.19 Plans for communicating important protocol amendments to relevant parties (e.g., trial participants, ethical committees)

If there are modifications to eligibility criteria, outcomes, or analyses needed for the study, a revised protocol will be submitted for approval to the Hospital Ethics Committee of the Second Rehabilitation Hospital of Shanghai, China.

### 2.20 Ethics and dissemination

The study protocol has been granted approval by the Ethics Committee of the Seventh People’s Hospital of Shanghai University of Traditional Chinese Medicine (approval number: 2024-7th-HIRB-093). The trial will be conducted in accordance with the principles set forth in the Declaration of Helsinki and will adhere to Good Clinical Practice standards. The informed consent form presented to participants comprehensively details the trial procedures, as well as the associated potential benefits and risks. No biological samples will be collected during this trial. The initiation of the study will occur only after participants have duly signed the informed consent form. In the event that significant modifications to the study protocol are required, these changes will be submitted for re-evaluation by the Ethics Committee.

## 3 Discussion

Upper limb motor dysfunction is a common and severe dysfunction in stroke patients, bringing a heavy burden on patients and their families, and an increasing demand for treatment and rehabilitation. At present, there are many neuromodulation techniques for motor dysfunction in stroke patients, including transcranial magnetic stimulation (TMS), (56–59) functional electric stimulation (FES), transcranial direct current stimulation (tDCS), (60, 61) etc. We hope to find a more effective and convenient treatment to improve the upper limb motor function in stroke patients.

This trial is one of the few clinical trials of a combination of ES and gamma oscillation in the treatment of upper limb motor dysfunction in stroke. One focus of this trial is that we will use ES, as widely used in post-stroke rehabilitation, to induce gamma oscillations. In clinical treatment, ES shows the advantages of simpler operation, safer use, more frequent application and more economical compared with other treatments such as TMS, FES and tDCS (62). If this study is successful, it will greatly improve the clinical treatment effect and benefit more stroke patients.

Another focus of this trial is that we will use comprehensive neurophysiological and clinical assessments to measure recovery of neurotransmission, upper limb movement, muscle coordination, and quality of life. The neurophysiological and clinical understanding of how this new neuromodulation method improves the motor function of the upper limb in post-stroke patients is extremely important to develop such neuromodulation intervention for motor dysfunction in stroke.

In conclusion, the present study aimed to investigate the effect of ES combined with gamma oscillations on the improvement of upper limb motor function in stroke patients. We hope that this innovative clinical trial will bring new enlightenment to the rehabilitation of stroke and provide strong support for improving the quality of life and reducing social and family burden. Of course, this still needs to be verified by a lot of research and clinical trials. Moreover, the short-term follow-up period of 2 weeks may inadvertently overlook the enduring implications of the intervention. While this brief timeframe can offer initial glimpses into immediate outcomes, it inadequately captures the nuanced and sustained impact that may unfold over an extended duration.

## Trial status

Recruitment began in October 2024 and the approximate date when recruitment will be completed in September 2025. The study protocol was approved by the ethical committees in September 2024.
