# Supplementary material for: The clinical efficacy and mechanism of gamma frequency electroacupuncture stimulation on the rehabilitation of upper limb motor function in stroke patients: study protocol of a randomized clinical trial
Source: Front Neurol. 2025 May 30;16:1603522. doi: 10.3389/fneur.2025.1603522 (PMC12162516; doi:10.3389/fneur.2025.1603522)
Supplement: Supplementary file 1 [file Data_Sheet_1.PDF]

Fugl-Meyer 评定量表

姓名：　　　　　　　性别：　　　　　年龄：　　　　　　　　填写医生：

上肢运动功能评定					
部位	运动功能检查	评分标准	评定日期<br>月 日	月 日	月 日
**上肢（坐位）**					
Ⅰ上肢反射活动	（1）肱二头肌腱反射	0 分：不能引出反射活动<br>2 分：能够引出反射活动			
	（2）肱三头肌腱反射				
Ⅱ屈肌共同运动	（1）肩关节上提	0 分：完全不能进行<br>1 分：部分完成<br>2 分：无停顿充分完成			
	（2）肩关节后缩				
	（3）外展（至少 90 度）				
	（4）外旋				
	（5）肘关节屈曲				
	（6）前臂旋后				
Ⅲ伸肌共同运动	（1）肩关节内收内旋	0 分：完全不能进行<br>1 分：部分完成<br>2 分：无停顿充分完成			
	（2）肘关节伸展				
	（3）前臂旋前				
Ⅳ伴有共同运动的活动	（1）手触腰椎	0 分：没有明显活动<br>1 分：手必须通过髂前上棘<br>2 分：能顺利进行			
	（2）肩关节屈曲 90 度（肘 关节 0 度时）	0 分：开始时手臂立即外展或肘关节屈曲<br>1 分：肩关节外展及肘关节屈曲发生较晚<br>2 分：能顺利充分进行			
	（3）在肩关节 0 度肘关节 90 度时前臂旋前或旋后	0 分：在进行该活动时肩关节 0 度但肘关节不能保持 90 度和完全不能完成该动作<br>1 分：肩关节正确位时能在一定范围内主动完成该动作<br>2 分：完全旋前或旋后活动自如			

Ⅴ分离运动	（1）肩关节外展 90 度时肘 关节 0 度位时前臂旋前	0 分：一开始时肘关节就屈曲、 前臂偏离方向不能旋前<br>1 分：可部分完成这个动作或者在活动时肘关节屈曲或前臂不能旋前<br>2 分：顺利完成			
	（2）肩关节屈曲度 90—180 度肘于 0 度位时前臂旋前旋 后	0 分：开始时肘关节屈曲或肩 关节外展发生<br>1 分：在肩部屈曲时，肘关节 屈曲，肩关节外展<br>2 分：顺利完成			
	（3）在肩关节屈曲 30 度— 90 度时，肘关节 0 度位时前 臂旋前或旋后	0 分：前臂旋前或旋后完全不能进行或肩肘位不正确<br>1 分：能在要求肢位时部分完成旋前旋后<br>2 分：顺利完成			
Ⅵ正常反射活动（该阶段者要得 2 分那么病人在第Ⅴ阶段必须得 6 分）	（1）肱二头肌腱反射	0 分：至少 2-3 个反射明显亢进<br>1 分：1 个反射明显亢进或至少 2 个反射活跃<br>2 分：反射活跃不超过1个并且无反射亢进			
	（2）指屈反射				
	（3）肱三头肌腱反射				
腕					
Ⅶ腕稳定性	（1）肘关节 90 度，肩关节 0 度	0 分：不能背屈腕关节达 15 度<br>1 分：可完成腕背屈，但不能抗阻<br>2 分：有些轻微阻力仍可保持腕背屈			
	（2）肘关节 90 度，肩关节 0 度时关节屈伸腕	0 分：不能随意运动<br>1 分：不能在全关节范围内活动腕关节<br>2 分：能平滑地不停顿地进行			
	（3）肘关节 0 度，肩关节 30 度	评分同（1）项			
	（4）肘关节 0 度，肩关节 30 度屈伸腕	评分同（2）项			
	（5）腕环行运动	0 分：不能进行<br>1 分：活动费力或不完全<br>2 分：正常进行			
手					
	（1）手指共同屈曲	0 分：不能屈曲<br>1 分：能屈曲但不充分<br>2 分：(与健侧比较）能完全主动屈曲			

Ⅷ手运动	（2）手指共同伸展	0 分：不能伸<br>1 分：能放松主动屈曲的手指<br>2 分：能充分主动的伸展			
	（3）握力 1：掌指关节伸展并且近端和远端指间关节屈曲，检测抗阻握力	0 分：不能保持要求位置<br>1 分：握力微弱<br>2 分：能够抵抗相当大的阻力 抓握			
	（4）握力 2：所有关节于 0 位时，拇指内收	0 分：不能进行<br>1 分：能用拇指捏住一张纸，但不能抵抗拉力<br>2 分：可牢牢捏住纸			
	（5）握力 3：患者拇食指可夹注一支铅笔	评分方法同握力 2			
	（6）握力 4：能握住一个圆筒物体	评分方法同握力 2、3			
	（7）握力 5：查握球形物体，如网球	评分方法同握力 2 、3 、4			
Ⅸ手协调性与速度：指鼻试验（快速连续进行 5 次）	（1）震颤	0 分：明显震颤<br>1 分：轻度震颤<br>2 分：无震颤			
	（2）辩距不良	0 分：明显的或不规则辩距障碍<br>1 分：轻度的规则的辩距障碍<br>2 分：无辩距障碍			
	（3）速度	0 分：较健侧慢 6 秒<br>1 分：较健侧慢 2-5 秒<br>2 分：两侧差别少于 2 秒			

上肢（共 33 项，各项最高分为 2 分，共 66 分）

下肢（共 17 项，各项最高分为 2 分，共 34 分）

运动功能积分：上肢_________ 下肢_________ 总分 _______________

Fugl-Meyer 运动功能评分的临床意义

**运动评分**	**分级**	**临床意义**
<50 分	I	严重运动障碍
50－84 分	II	明显运动障碍
85－95 分	III	中度运动障碍
96－99 分	IV	轻度运动障碍
