# Supplementary material for: The clinical efficacy and mechanism of gamma frequency electroacupuncture stimulation on the rehabilitation of upper limb motor function in stroke patients: study protocol of a randomized clinical trial
Source: Front Neurol. 2025 May 30;16:1603522. doi: 10.3389/fneur.2025.1603522 (PMC12162516; doi:10.3389/fneur.2025.1603522)
Supplement: Supplementary file 2 [file Data_Sheet_2.PDF]

美国国立卫生研究院卒中量表（NIHSS）

姓名： **性别：** **年龄：** **填写医生：**

检查项目	评分标准	评分		
		月<br>日	月<br>日	月<br>日
1（a）意识水平：<br>即使不能全面评价（如气管插管、语言障碍、气管创伤、绷带包扎等），检查者也必须选择 1 个反应。只在病人对有害刺激无反应时（不是反射），方记录 3 分。	0＝清醒，反应敏锐<br>1＝嗜睡，最小刺激能唤醒病人完成指令、回答问题或有反应<br>2＝昏睡或反应迟钝，需要强烈反复刺激或疼痛刺激才能有非固定模式的反应<br>3＝仅有反射活动或自发反应，或完全没反应、软瘫、无反应			
1（b）意识水平提问：（仅对最初回答评分，检查者不要提示）<br>询问月份，年龄。回答必须正确，不能大致正常。失语和昏迷者不能理解问题记 2 分，病人因气管插管、气管创伤、严重构音障碍、语言障碍或其他任何原因不能说话者（非失语所致）记 1 分。可书面回答。	0＝都正确<br>1＝正确回答一个<br>2＝两个都不正确或不能说			
1（c）意识水平指令：<br>要求睁眼、闭眼：非瘫痪手握拳、张手。若双手不能检查，用另一个指令（伸舌）。仅对最初的反应评分，有明确努力但未完成也给评分。若对指令无反应，用动作示意，然后记录评分。对创伤、截肢或其他生理缺陷者，应给予一个适宜的指令。	0＝都正确<br>1＝正确完成一个<br>2＝都不正确			
2 凝视：<br>只测试水平眼球运动。对自主或反射性（眼头）眼球运动记分。若眼球侧视能被自主或反射性活动纠正，记录 1 分。若为孤立性外周神经麻痹（Ⅲ、Ⅳ、Ⅴ），记 1 分。在失语病人中，凝视是可测试的。对眼球创伤、绷带包扎、盲人或有	0＝正常<br>1＝部分凝视麻痹（单眼或双眼凝视异常，但无被动凝视或完全凝视麻痹）<br>2＝ 被动凝视或完全凝视麻痹（不能被眼头动作克服）			

视觉或视野疾病的患者，由检查者选择一种反射性运动来测试。建立与眼球的联系，然后从一侧向另一侧运动，偶尔能发现凝视麻痹。				
3 视野：<br>用手指数或视威胁方法检测上、下象限视野。如果病人能看到侧面的手指，记录正常。如果单眼盲或眼球摘除，检查另一只眼。明确的非对称盲（包括象限盲），记 1 分。病人全盲（任何原因）记 3 分，同时刺激双眼。若人濒临死亡记 1 分，结果用于回答问题 11。	0＝无视野缺失<br>1＝部分偏盲<br>2＝完全偏盲<br>3＝双侧偏盲（全盲，包括皮质盲）			
4 面瘫：<br>言语指令或动作示意，要求病人示齿、扬眉和闭眼。对反应差或不能理解的病人，根据有害刺激时表情的对称情况评分。有面部创伤/绷带、经口气管插管、胶布或其他物理障碍影响面部检查时，应尽可能移至可评估的状态。	0＝正常<br>1＝最小（鼻唇沟变平、微笑时不对称）<br>2＝部分（下面部完全或几乎完全瘫痪，中枢性瘫）<br>3＝完全（单或双侧瘫痪，上下面部缺乏运动，周围性瘫）			
5 上肢运动：<br>上肢伸展：坐位 90º，位卧 45º。要求坚持 10 秒；对失语的病人用语言或动作鼓励，不用有害刺激。评定者可以抬起病人的上肢到要求的位置，鼓励病人坚持。仅评定患侧。	0＝上肢于要求位置坚持 10 秒无下落<br>1 ＝上肢能抬起，但不能维持 10 秒，下落时不撞击床或其他支持物<br>2＝能对抗一些重力，但上肢不能达到或维持坐位 90º 或位卧 45º，较快下落到床上<br>3＝不能抗重力，上肢快速下落<br>4＝无运动<br>9＝截肢或关节融合，解释：<br>5a 左上肢 5b 右上肢			
6 下肢运动：<br>下肢卧位抬高 30º，坚持 5 秒；对失语的病人用语言或动作鼓励，不用有害刺激。评定者可以抬起病人的上肢到要求的位置，鼓励病人坚持。仅评定患侧。	0＝于要求位置坚持 5 秒，不下落<br>1＝在 5 秒末下落，不撞击床<br>2＝5 秒内较快下落到床上，但可抗重力<br>3＝快速落下，不能抗重力<br>4＝无运动<br>9＝截肢或关节融合，解释：<br>6a 左下肢 6b 右下肢			

7 共济失调：<br>目的是发现双侧小脑病变的迹象。实验时双眼睁开，若有视觉缺损，应确保实验在无缺损视野内进行。双侧指鼻、跟膝胫试验，共济失调与无力明显不呈比例时记分。如病人不能理解或肢体瘫痪不记分。盲人用伸展的上肢摸鼻。若为截肢或关节融合，记录 9 分，并解释清楚。	0＝没有共济失调<br>1＝一个肢体有<br>2＝两个肢体均有<br>如有共济失调：<br>右上肢 1＝是 2＝否<br>9＝截肢或关节融合，解释：<br>左上肢 1＝是 2＝否<br>9＝截肢或关节融合，解释：<br>右上肢 1＝是 2＝否<br>9＝截肢或关节融合，解释：<br>左下肢 1＝是 2＝否<br>9＝截肢或关节融合，解释：<br>右下肢 1＝是 2＝否			
8 感觉：<br>用针检查。测试时，用针尖刺激和撤除刺激观察昏迷或失语病人的感觉和表情。只对与卒中有关的感觉缺失评分。偏身感觉丧失者需要精确检查，应测试身体多处部位：上肢（不包括手）、下肢、躯干、面部。严重或完全的感觉缺失，记 2 分。昏迷或失语者可记 1 或 0 分。脑干卒中双侧感觉缺失记 2 分。无反应及四肢瘫痪者记 2 分。昏迷病人（1a＝3）记 2 分。	0＝正常，没有感觉缺失<br>1＝轻到中度，患侧针刺感不明显或为钝性或仅有触觉<br>2＝严重到完全感觉缺失，面、上肢、下肢无触觉			
9 语言：<br>命名、阅读测试。要求病人叫出物品名称、读所列的句子。从病人的反应以及一般神经系统检查中对指令的反应判断理解能力。若视觉缺损干扰测试，可让病人识别放在手上的物品，重复和发音。气管插管者手写回答。昏迷病人（1a＝3），3 分,给恍惚或不合作者选择一个记分，但 3 分仅给不能说话且不能执行任何指令者。	0＝正常，无失语<br>1＝轻到中度：流利程度和理解能力有一些缺损,但表达无明显受限。<br>2＝严重失语，交流是通过病人破碎的语言表达，听者须推理、询问、猜测,能交换的信息范围有限，检查者感交流困难。<br>3＝不能说话或完全失语，不能讲或不能理解			
10 构音障碍：<br>不要告诉病人为什么做测试。<br>读或重复附表上的单词。若病人有严重的失语，评估自发语言时发音的清晰度。若病人气管插管或其他物理障碍不能讲话，记 9 分。	0＝正常<br>1＝轻到中度，至少有一些发音不清，虽有困难，但能被理解<br>2＝言语不清，不能被理解<br>9＝气管插管或其他物理障碍，			

11 忽视症：<br>若病人严重视觉缺失影响双侧视觉的同时检查，皮肤刺激正常，则记分为正常。若病人失语，但确实表现为关注双侧，记分正常。<br>通过检验病人对左右侧同时发生的皮肤感觉和视觉刺激的识别能力来判断病人是否有忽视。把标准图显示给病人，要求他来描述。医生鼓励病人仔细看图，识别图中左右侧的特征。如果病人不能识别一侧图的部分内容，则定为异常。然后，医生请病人闭眼，分别测上或下肢针刺觉来检查双侧皮肤感觉。若病人有一侧感觉忽略则为异常。	0＝没有忽视症<br>1＝视、触、听、空间觉或个人的忽视;或对任何一种感觉的双侧同时刺激消失<br>2＝严重的偏身忽视；超过一种形式的偏身忽视;不认识自己的手，只对一侧空间定位			
**总分**				
