# Supplementary material for: The clinical efficacy and mechanism of gamma frequency electroacupuncture stimulation on the rehabilitation of upper limb motor function in stroke patients: study protocol of a randomized clinical trial
Source: Front Neurol. 2025 May 30;16:1603522. doi: 10.3389/fneur.2025.1603522 (PMC12162516; doi:10.3389/fneur.2025.1603522)
Supplement: Supplementary file 3 [file Data_Sheet_3.PDF]

简易认知状态评价量表（MMSE）

姓名：　　　　　　性别：　　　年龄：　　　受教育水平：　　　　填写医生：

项目		积分					
定向力<br>（10分）	1.今年是哪一年<br>现在是什么季节?<br>现在是几月份?<br>今天是几号?<br>今天是星期几?					1<br>1<br>1<br>1<br>1	0<br>0<br>0<br>0<br>0
	2.你住在那个省?<br>你住在那个县（区）?<br>你住在那个乡（街道）?<br>咱们现在在那个医院?<br>咱们现在在第几层楼?					1<br>1<br>1<br>1<br>1	0<br>0<br>0<br>0<br>0
记忆力<br>（3分）	3.告诉你三种东西，我说完后，请你重复一遍并记住，待会还会问你（各1分，共3分）			3	2	1	0
注意力和计算力<br>（5分）	4.100-7=?连续减 5 次（93、86、79、72、65。各 1 分，共5分。若错了，但下一个答案正确，只记一次错误）	5	4	3	2	1	0
回忆能力<br>（3分）	5.现在请你说出我刚才告诉你让你记住的那些东西?			3	2	1	0
语言能力<br>(9分)	6. 命名能力<br>出示手表，问这个是什么东西?<br>出示钢笔，问这个是什么东西?					 1<br>1	 0<br>0
	7. 复述能力<br>我现在说一句话，请跟我清楚的重复一遍（四十四只石狮子）					1	0
	8.阅读能力<br>（闭上你的眼睛）请你念念这句话，并按上面意思去做					1	0
	9.三步命令<br>我给您一张纸请您按我说的去做，现在开始：“用右手拿着这张纸，用两只手将它对折起来，放在您的左腿上。”（每个动作1分，共3分）			3	2	1	0
	10.书写能力要求受试者自己写一句完整的句子					1	0
	11.结构能力<br>（出示图案）请你照上面图案画下来					1	0

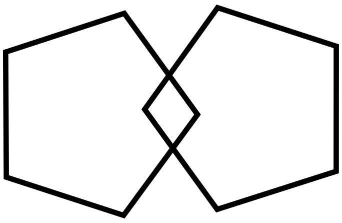

<table>
<tr><th colspan="5">视空间与执行功能</th></tr>
<tr><td rowspan="3">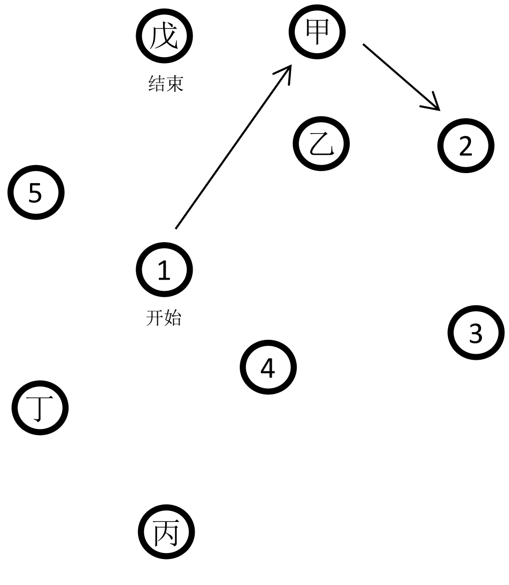
</td><td rowspan="2">复制立方体</td><td colspan="3">画钟表（11点30分）（3分）</td></tr>
<tr><td colspan="3"></td></tr>
<tr><td>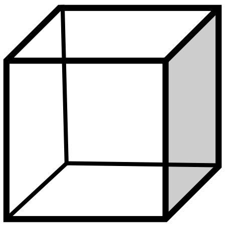</td><td>轮廓</td><td>数字</td><td>指针</td></tr>
</table>

评估时间：______________________

评估阶段：□初　　□中　　□末

总分：______________________

注：最高得分为 30 分，分数在 27-30 分为正常,分数 <27 为认知功能障碍。
