# Supplementary material for: The clinical efficacy and mechanism of gamma frequency electroacupuncture stimulation on the rehabilitation of upper limb motor function in stroke patients: study protocol of a randomized clinical trial
Source: Front Neurol. 2025 May 30;16:1603522. doi: 10.3389/fneur.2025.1603522 (PMC12162516; doi:10.3389/fneur.2025.1603522)
Supplement: Supplementary file 4 [file Data_Sheet_4.PDF]

改良 Barthel 指数评分标准

姓名：　　　　　　性别：　　　　　　年龄：　　　　　　填写医生：

项　目	评分标准	评分		
		月　　日	月　　日	月　　日
1 进食	完全独立 10<br>少量帮助 8<br>中等帮助 5<br>大量帮助 2<br>完全依赖 0			
2. 洗澡	完全独立 5<br>少量帮助 4<br>中等帮助 3<br>大量帮助 2<br>完全依赖 1			
3. 修饰	完全独立 5<br>少量帮助 4<br>中等帮助 3<br>大量帮助 2<br>完全依赖 1			
4. 穿衣	完全独立 10<br>少量帮助 8<br>中等帮助 5<br>大量帮助 2<br>完全依赖 0			
5. 大便控制	完全独立 10<br>少量帮助 8<br>中等帮助 5<br>大量帮助 2<br>完全依赖 0			
6. 小便控制	完全独立 10<br>少量帮助 8<br>中等帮助 5<br>大量帮助 2<br>完全依赖 0			
7. 如厕	完全独立 10<br>少量帮助 8<br>中等帮助 5<br>大量帮助 2<br>完全依赖 0			
8. 转移	完全独立 15<br>少量帮助 12<br>中等帮助 8<br>大量帮助 3<br>完全依赖 0			

9．行走	完全独立 15<br>少量帮助 12<br>中等帮助 8<br>大量帮助 3<br>完全依赖 0			
10．轮椅操作	完全独立 5<br>少量帮助 4<br>中等帮助 3<br>大量帮助 2<br>完全依赖 0			
11．上下楼梯	完全独立 10<br>少量帮助 8<br>中等帮助 5<br>大量帮助 2<br>完全依赖 0			
总　分				

基本的评级标准:每个活动的评级可分 5 级（5 分），不同的级别代表了不同程度的独立能力，最低的是 1 级，而最高是 5 级。级数越高，代表独立能力越高。
