# Supplementary material for: The clinical efficacy and mechanism of gamma frequency electroacupuncture stimulation on the rehabilitation of upper limb motor function in stroke patients: study protocol of a randomized clinical trial
Source: Front Neurol. 2025 May 30;16:1603522. doi: 10.3389/fneur.2025.1603522 (PMC12162516; doi:10.3389/fneur.2025.1603522)
Supplement: Supplementary file 5 [file Data_Sheet_5.PDF]

上海市第七人民医院伦理委员会伦理审查批件

批件号：2024-7th-HIRB - 093

**研究名称**	40Hz 电针刺激诱发伽马神经振荡促进脑卒中患者上肢运动功能康复的临床疗效及作用机制探索		
**申办单位**	上海市第七人民医院		
**主要研究者**	于小明	**研究者科室**	康复治疗科
**审查类别**	复审审查	**审查方式**	快速审查
**伦理委员会审议成员**	金珠、雷鸣		
**伦理委员会地址**	上海市浦东新区高桥镇大同路 358 号 1 号楼辅楼 203 室		
**审议时间**	2024 年 8 月 28 日		
**审查材料**	1. 复审审查申请表单<br>2. 研究方案（版本号：02，版本日期：2024 年 8 月 23 日）<br>3. 知情同意（版本号：02，版本日期：2024 年 8 月 23 日）<br>4. 团队组成表		
**审议结论**	根据最新版《药物临床试验质量管理规范》、《药物临床试验伦理审查工作指导原则》、《涉及人的生物医学研究伦理审查办法》以及《赫尔辛基宣言》和国际医学科学组织委员会颁布的《人体生物医学研究国际道德指南》的道德原则。本伦理委员会审阅并讨论了以上材料，经投票表决**批准**你们自即日起开展该临床试验研究，并要求：<br>（1）上述材料未经本伦理委员会批准，不得作任何修改；<br>（2）发生严重不良事件，请研究者获知 24h 内提交严重不良事件报告；<br>（3）请根据跟踪审查频率并在批件失效前 1 个月递交跟踪审查报告；<br>（4）如出现违背方案的情况，请递交违背方案报告；<br>（5）如研究结束，请及时递交研究结题报告。		
**副主任委员签字**			
**主任委员签字**			
**跟踪审查频率**	3 个月　6 个月　9 个月　12 个月	**联系电话**	021-58670561-6659/6449
**批件有效期**	一年		

上海市第七人民医院

伦理委员会（盖章）

2024 年 9 月 3 日
