# Supplementary material for: The clinical efficacy and mechanism of gamma frequency electroacupuncture stimulation on the rehabilitation of upper limb motor function in stroke patients: study protocol of a randomized clinical trial
Source: Front Neurol. 2025 May 30;16:1603522. doi: 10.3389/fneur.2025.1603522 (PMC12162516; doi:10.3389/fneur.2025.1603522)
Supplement: Supplementary file 6 [file Data_Sheet_6.pdf]

# Ethical review approval of the Seventh Peoples Hospital of Shanghai

Approval number: 2024-7th-HIRB-093

| | | | |
|---|---|---|---|
| Name of the study | The clinical efficacy and mechanism of gamma oscillation induced by 40Hz electro-stimulation in promoting the rehabilitation of upper limb motor function in stroke patients were explored | | |
| Applicant | Shanghai Seventh Peoples Hospital | | |
| Lead researcher | Yu Xiaoming | Researcher department | Rehabilitation Department |
| Review categories | Review | Methods of review | Rapid review |
| Ethical committee members | Jin Zhu, Leiming | | |
| Address of ethics committee | No.203, auxiliary building, Building 1, No.358 Datong Road, Gaoqiao Town, Pudong New Area, Shanghai | | |
| Considerati on time | August 28,2 0 2 4 | | |
| Review materials | 1.Application form for review examination<br>2.Research Program (Version No. 02, Version Date: August 23, 2024)<br>3.Informed Consent Form (Version No. 02, Version Date: August 23, 2024)<br>4.Table of Team Composition | | |
| Conclusions of the review | According to the latest edition of the Good Clinical Practice for Drug Clinical Trials, the Guidelines for Ethical Review of Drug Clinical Trials, the Ethics Review Measures fo Biomedical Research Involving Humans, and the ethical principles outlined in the Declaratio of Helsinki and the International Council for Medical Sciences International Code of Ethic for Human Biomedical Research, this ethics committee has reviewed and discussed the abov materials, and upon voting, approves your initiation of this clinical trial research fro this date onward, with the following requirements:<br>(1) No modification shall be made to the above materials without the approval of this ethics committee;<br>(2) In case of serious adverse events, the investigator shall submit a serious adverse event report within 24 hours;<br>(3) Please submit the follow-up review report according to the frequency of follow-up review and one month before the expiration of the approval document;<br>(4) If there is any violation of the plan, please submit a report of violation; | | |

| | | | |
|---|---|---|---|
| | 5) If the study is completed, please submit the research conclusion report in time. | | |
| Signature of the Deputy Chairman | | | |
| Signature of the Chairperson | | | |
| Tracking review frequency | 3 months 6 months 9 months 12 months | Contact details | 021-58670561-6659/6449 |
| Validity period of the approval document | one year | | |

Shanghai Seventh Peoples Hospital

Ethical Committee (stamped)

September 3, 2024
